# Pancreas Imaging of Children with Type 1 Diabetes Reveals New Patterns and Correlations with Pancreatic Functions

**DOI:** 10.1155/2023/3295812

**Published:** 2023-09-22

**Authors:** Olivier G. Pollé, Antoine Delfosse, Nicolas Michoux, Frank Peeters, Gaetan Duchêne, Jacques Louis, Brieuc Van Nieuwenhuyse, Philippe Clapuyt, Philippe A. Lysy

**Affiliations:** ^1^Pôle PEDI, Institut de Recherche Expérimentale et Clinique, UCLouvain, Brussels, Belgium; ^2^Specialized Pediatrics Service, Cliniques Universitaires Saint-Luc, Brussels, Belgium; ^3^Department of Radiology, Institut de Recherche Expérimentale et Clinique, Cliniques Universitaires Saint Luc, UCLouvain, Brussels, Belgium; ^4^MR Applications, General Electric Healthcare, Diegem, Belgium; ^5^Division of Pediatric Endocrinology, Department of Pediatrics, Grand Hôpital de Charleroi, Charleroi, Belgium

## Abstract

**Objective:**

To perform a longitudinal characterization of the pancreas in patients with new-onset T1D and investigate the correlations between magnetic resonance imaging (MRI) parameters and pancreatic functions during the first year postdiagnosis.

**Methods:**

Thirty-one pediatric patients with new-onset T1D and 29 retrospective age-, body mass index-, and sex-matched controls were included in the study. Following hypotheses were investigated: (H1) the value of pancreas volume (PV) parameters in T1D and in controls, (H2) the association between MRI parameters and markers of pancreatic functions, (H3) the ability of MRI parameters to predict glucose homeostasis, (H4) the longitudinal evolution of MRI parameters and glucose homeostasis, *per*-organ (whole pancreas) and *per*-subregion (head, body, and tail).

**Results:**

Patients with new-onset T1D demonstrated a significant decrease of PV at diagnosis compared to controls (−45%), with prepubertal patients having increased pancreas atrophy (+25%) (H1). PV parameters were correlated with C-peptide, and trypsinogen (PV_Tail_ and PV_Head_, respectively). Biparametric regression models including MRI parameters predicted pancreas functions during the first year postdiagnosis (H3). Longitudinal evolution of PV parameters at 1 year postdiagnosis was correlated with PV at diagnosis (*R* = −0.72) but not with markers of glucose homeostasis (H4).

**Conclusion:**

Our study shows that longitudinal analysis of pancreases of children with T1D using multiparametric MRI improve the understanding of T1D heterogeneity both in the context of its onset and its evolution.

## 1. Introduction

One of the most predominant challenges in modern medicine is the clinically relevant characterization of disease heterogeneity between patients, with the perspective of personalization of care. Recent studies support the existence of multiple clinical entities in patients with type 1 diabetes (T1D) that show distinct clinical and histological characteristics. However, biomarkers to distinguish these subgroups currently lack and lead to a poor integration of the disease patchiness in the clinical routine and major barriers in the translational research.

T1D is one of the most prevalent chronic autoimmune diseases during childhood, which is characterized by the progressive destruction of insulin-secreting *β* cells. However, major questions remain unsolved; especially the nature of immune attack (e.g., autoimmune process or *β*-cell vulnerability) [1], the extent of disease heterogeneity (in terms of clinical onset and evolution) [2], and the presence of biomarkers that allow the prediction of residual endocrine function evolution [3–5]. Interactions between *β* and acinar cells were also proposed as key elements in T1D pathogeny, renewing the hypothesis of T1D as a whole-pancreas disease [6, 7].

Although *β* cells only represent ∼2% of the total pancreatic mass, it is known for more than a century that patients with T1D present both a reduction of half of their pancreas weight and pancreatic exocrine dysfunction [8, 9]. Recent studies showed that the reduction of pancreas volume (PV) is already present in the earliest stages of T1D, with presymptomatic islet-autoantibodies (IAb) positive individuals having intermediate levels of pancreatic volume and decreased exocrine function (i.e., between new-onset T1D and control patients) [10, 11]. Some first-degree relatives of patients with T1D also developed autoantibodies against acinar cells (e.g., anti-BDSL) before progressing with anti-islet autoimmunity and finally overt T1D [12]. Finally, up to 70% of patients with T1D had circulating autoantibodies against the exocrine tissue, supporting a global immune attack of the pancreas in a part of the individuals [13, 14]. Nonetheless, the exact temporality (before, concomitantly, or after *β*-cell demise) and mechanisms underlying the structural modifications and dysfunction of the exocrine pancreas remain largely unknown. Finally, both clinical manifestations and the extent of exocrine insufficiency vary among patients with T1D [9, 10, 15].

Endotypes (i.e., groups of individuals with specific histological, genetic and clinical features) were recently described in patients with new-onset T1D and suggested distinct etiopathological mechanisms [1, 16–18]. These endotypes showed age-related differences (<7 years old or >12 years old) with young patients demonstrating a more aggressive T1D pattern characterized by higher incidence of high-risk genotypes [18], increased markers of inflammation and immunity at T1D onset [19], and a steeper decline of residual *β*-cell secretion in the weeks following T1D diagnosis [20]. While histological distinctions between endotypes mostly relied on the endocrine compartment (e.g., immune islet infiltration and insulin processing) [16, 17], differences have not yet been investigated for exocrine features.

Magnetic resonance imaging (MRI) is a cardinal tool in the evaluation of pancreatic pathologies as it allows real-time and *in situ* quantification of multiple tissue parameters (e.g., volume, structural modifications, tissue diffusion, fat disposal) [21]. Corroborating previous anatomical and histological descriptions, MRI of patients with T1D showed a reduction of PV, alterations of pancreas microvasculature and a patchy infiltration of the whole organ [15, 22]. MRI also proved to be minimally invasive, cost-effective, and repeatable in the context of longitudinal pediatric studies [23–25]. Few MRI studies investigated pediatric T1D cohorts and mostly focused on the evaluation of a single MRI parameter, the PV. However, no clear trend appeared in the correlation between these imaging features, and both exocrine and endocrine functions [10, 15, 26–29]. The incomplete characterization of patient phenotype (in terms of cross-sectional measurements of both endocrine and exocrine functions) and the lack of topographic measurements of pancreatic parameters may explain this discrepancy between the studies.

The purpose of this subsidiary study of the DIATAG cohort is to perform a topographic and phenotypic characterization of the pancreas in pediatric patients with new-onset T1D. Univariate and multiparametric approaches to assess the association between pancreas MRI parameters, and exocrine and endocrine pancreatic functions were performed upon diagnosis (baseline study) and after 12 months (longitudinal study).

## 2. Materials and Methods

### 2.1. Study Design

This study included a subset of participants from the Belgian multicentric DIATAG cohort. The aim of DIATAG trial was to characterize the heterogeneity of *β*-cell function evolution in pediatric patients with new-onset T1D during the first year after clinical onset. Inclusion and exclusion criteria were described elsewhere (NCT04007809). T1D was diagnosed using ISPAD criteria [30] which included at least one circulating anti-islet autoantibody (anti-insulin, anti-protein tyrosine phosphatase, anti-glutamic acid decarboxylase or, anti-Zinc transporter 8). All participants and their parents gave their written consent prior to the enrollment in the study. The protocol was approved by the principal ethical committee (Comité d'Ethique Hospitalo-Facultaire of Cliniques universitaires Saint-Luc (CUSL), [2018/04DEC/462]) and local ethical committees of all participating institutions. The trial was registered at www.clinicaltrial.gov.

### 2.2. Study Procedures

The baseline screening included the patient demographics at diagnosis (*Δ*) and a blood draw that was performed after an overnight fast between *Δ* + 5 and *Δ* + 21 days. Pubertal status was determined during the initial hospitalization either by pediatric endocrinologists (Tanner stage) or serum LH levels when clinical examination was borderline (e.g., early M2 or testicular volume 4–6 mL) or absent. Participants were either classified as prepubertal (Tanner I or LH < 0.3 UI/L) or pubertal (Tanner II-V or LH > 0.3 UI/L). After the initial hospitalization, the outpatient clinical follow-up in diabetes care conventions was organized throughout routine visits at *Δ* + 3, *Δ* + 6, *Δ* + 9, and *Δ* + 12 months as previously described [31]. During these visits, residual *β*-cell secretion and routine parameters (i.e., total insulin daily dose [TDD], insulin dose-adjusted A1_C_ [IDAA_1C_], HbA_1C_) were evaluated. Data from patients' medical records were gathered and registered inside the Research Electronic Data Capture (REDCap) system [32].

### 2.3. Measure of Residual *β*-Cell Secretion

Sample collection and assay characteristics were previously described [31]. Estimated residual stimulated C-peptide (CPEP_EST_) was evaluated at *Δ* + 3 months and *Δ* + 12 months using a mathematical formula that integrates both biological measures before stimulation test (i.e., CPEP_BASAL_ (fasting C-peptide), fasting plasma glucose, HbA_1C_) and clinical parameters (i.e., body mass index (BMI), TDD, and disease duration) [33]. In this study, CPEP_EST_ data were used as they were available for most patients and demonstrated strong correlations with stimulated C-peptide with both glucagon stimulation tests [31] and mixed-meal tolerance test [34].

### 2.4. Remission Status

Remission status was determined using the IDAA_1C_ score, as follows: HbA_1C_ (%) + (4 × insulin dose (U/kg body weight per 24 hr) [35], with a score below 9 defining the occurrence of remission. TDD was either reported by patients (multiple daily injection users) or calculated using the software for pump users.

### 2.5. Pancreatic Exocrine Function

The exocrine function was evaluated by measuring serum immunoreactive trypsinogen levels on fasting samples at baseline. Serum immunoreactive trypsinogen levels were determined using radioimmunoassay (KIPCE07, DIAsource ImmunoAssays S.A., Louvain-la-Neuve, Belgium) at CUSL central laboratory, with a normal laboratory reference ranging from 140 to 400 ng/mL and inter- and intra-assay coefficients of variation, respectively, ranging from 4.9% to 6.7% and from 2.8% to 3.3%.

### 2.6. Multiparametric Pancreas MRI Assessment

Participants aged more than 5 years (<100 days after diagnosis) underwent a pancreas MRI without injection or sedation at CUSL. Participants were not fasted before imaging [15, 23]. Clinical metrics (weight in kg and height in cm) were collected on the day of the MRI scan or from a recent medical visit (<1 month, >30 days after diagnosis). MRI was repeated for a subset of participants after a minimum of 10 months to allow the longitudinal assessment of quantitative parameters (see below).

### 2.7. Pancreas MRI

Pancreas imaging was performed on a 3T Signa Premier MRI scanner (GE Healthcare, Milwaukee, WI, USA). Axial MR images were acquired from inferior to superior, until the whole PV was covered. Parameters of the MRI sequences are given in Table S1.

Anatomical characterization was performed using a 2D multislice T2-weighted Single-Shot Fast Spin Echo (T2w-SSFSE) sequence and a 3D T1-weighted SPoiled GRadient (T1w-SPGR, LAVA Flex) echo sequence.

Diffusion weighted imaging (DWI) was performed with a single-shot echo planar imaging (EPI) sequence with diffusion encoding applied in three orthogonal directions for three *b*-values (0, 50, and 800 s/mm^2^) [36]. In order to minimize the contribution of the perfusion, only the two largest *b*-values were used to compute the ADC (apparent diffusion coefficient).

Fat quantification was performed using a 3D Multi-echo GRadient Echo (Multi-echo GRE, IDEAL-IQ) sequence with a 3° flip angle for fat quantification. Six equidistant echoes were acquired (echo times TE from 0.9 to 4.3 ms) to reconstruct the in-phase, out-of-phase, water (W), fat (F), and R2*∗* = 1/T2*∗* maps with the manufacturer's algorithm IDEAL-IQ.

All ADC maps and fat-fraction (FF, F/(F + W)) maps were computed offline by MRI physicists using in-house software under Matlab (Matlab R2011b, MathWorks, Natick, MA, USA).

### 2.8. Segmentation and Volumetry of Pancreas

Segmentation of the whole pancreas and of the pancreas subregions (from which PV was derived) was performed by three readers (O.P., A.D., and P.C.) as follows: the pancreas was manually delineated on each imaging slice from the water-only series of the T1w-SPGR sequence using an offline image processing workstation (Vitrea FX, Vital Images, Minnetonka, MN, USA). T2w-SSFSE images were used when the pancreas could not be accurately delineated on T1w-SPGR images. When interobserver differences between PV measures exceeded 10%, images were reviewed in consensus.

Pancreas subregions were defined as follows: head, body, and tail. The head was defined as the organ subregion located from the duodenum to the left border of the superior mesenteric vein. The body was defined as the subregion downstream of the pancreas head to the median border of the left kidney. The tail was defined as the subregion that lies anteriorly to the left kidney, from its median border to the splenic hilum (Figure S1) [37–39].

The PV was automatically generated by the imaging processing workstation. The pancreas volume index (PVI) was calculated by dividing the PV by the weight of the participant during the first outpatient visit (i.e., baseline study) [10] or at 12-month visit (i.e., longitudinal study). Pancreas volume ratio (PVR) was computed to investigate the topography of PV reduction and calculated as follows:

(1) $$\begin{array}{c} \text{PVR}=\frac{\text{PV of the tail}+\text{PV of the body}}{\text{PV of the head}}. \end{array}$$

To classify patients with T1D according to PV parameters at 12 months, PVI ratio was calculated as PVI^T12^/PVI^T0^. Longitudinal evolution and association between MRI and clinical parameters was assessed in terms of absolute differences (Parameter^T0^−Parameter^T12^) and relative differences ((Parameter^T0^−Parameter^T12^)/Parameter^T0^).

### 2.9. Controls

Controls were retrospectively identified using the Picture Archiving and Communication Systems (PACS) database in the participating centers (CUSL, Grand hôpital de Charleroi and CHC MontLégia) and matched with diabetic participants for age, sex, and BMI. Medical records were screened by a board-certified attending pediatrician (O. P.) to exclude patients with history of pancreatic disease (either pancreatic masses, pancreatic trauma, medical history of pancreatitis, ciliopathy, or cystic fibrosis) and/or treatment with pancreas toxicity (radiotherapy and chemotherapy) [40]. MRI scans with incomplete PV or blurred pancreatic outlines were excluded from the analysis.

Volume measurements were performed identically to the T1D cohort. The reading physician was not blinded to the patient condition to identify the possible confounding images and improve the accuracy of delineation.

### 2.10. Statistical Analysis

Data analysis was performed using Matlab and R (R Core Team) software. A *p*-value < 0.05 was regarded as statistically significant for all analyses.

Demographic and clinical data were reported as the mean ± SD for continuous variables and as numbers and proportions for categorical variables. A two-sided *t*-test (equivalently a two-sided Mann–Whitney *U* test when data distributions were nonnormal according to the Shapiro–Wilk test) was used to assess differences in group means (respectively, in group medians). Differences in proportions were assessed using chi-square or Fisher exact test as appropriate. A paired *t*-test (equivalently a Wilcoxon test) was used to assess mean (equivalently median) differences over time within a same group (longitudinal study). A four-way ANOVA with fixed effects was performed to assess the influence of the factors “disease status,” “sex,” “pubertal stage,” and “anatomical subregions” on MRI parameters (PV, PVI, and PVR).

Correlations between PV parameters and pancreatic functions markers were assessed using Spearman's correlation coefficient.

Multiparametric linear regression models were implemented to assess whether MRI parameters (measured from the whole pancreas, head, body, or the tail) at diagnosis predicted endocrine functions of the patient at *Δ* + 3, *Δ* + 6, *Δ* + 9, and *Δ* + 12 months postdiagnosis. A backward procedure (significance level to enter the parameters into the model: *p* < 0.20, significance level to remove them: *p* > 0.04) was applied to select the independent parameters. Then the multiple correlation coefficient *R* of each model was reported.

## 3. Results

### 3.1. Population

Demographics are summarized in Table 1. The study included 31 pediatric patients with new-onset T1D from the DIATAG trial and 29 retrospectively identified nondiabetic controls matched for age, sex, BMI, and pubertal status. Patients with new-onset T1D underwent an initial MRI scan at a mean ± SD age of 11 ± 2.9 years, shortly after clinical diagnosis (mean duration = 40 ± 37 days).

### 3.2. Study Hypotheses

**Figure 1:**
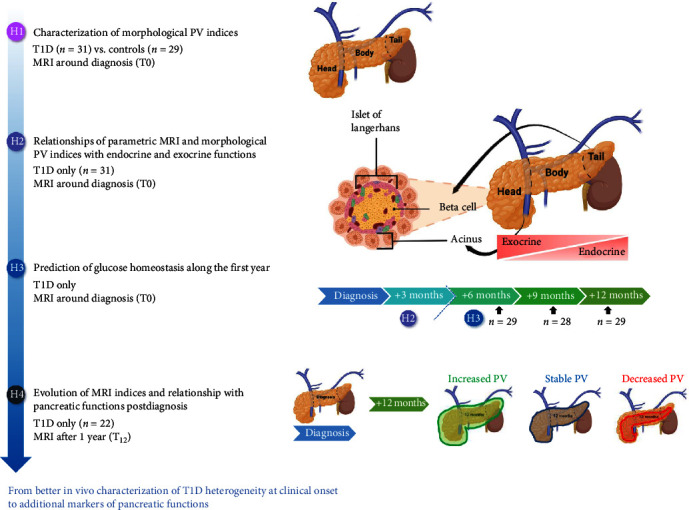
Flowchart of the DIATAG MRI study. The following hypotheses were investigated in the study. H1: analysis performed on anatomical MRI imaging (PV, PVI, and PVR) according to the disease status, the pancreas subregions and the pubertal status (T0). H2: analysis performed on anatomical, diffusion and FAT MRI (T0). Exocrine (i.e., trypsine) and endocrine (CPEP_EST_, IDAA_1C_, HbA_1C_, and TDD) functions were evaluated around diagnosis. H3: analysis performed on anatomical, diffusion and FAT MRI (T0). Exocrine (i.e., trypsine) and endocrine (CPEP_EST_, IDAA_1C_, HbA_1C_, and TDD) were evaluated at *Δ* + 3? months, *Δ* + 6, *Δ* + 9, and *Δ* + 12 months. H4: analysis performed on anatomical, diffusion and fat MRI (T12). Exocrine (i.e., trypsine) and endocrine (CPEP_EST_, IDAA_1C_, HbA_1C_, and TDD) were evaluated at *Δ* + 12 months. Abbreviations: H, hypothesis; MRI, magnetic resonance imaging; PV, pancreatic volume.

A flowchart of the hypotheses investigated in the study is provided in Figure 1. Results presented thereafter are organized according to this flowchart. Principal results were summarized in Table 2.

#### 3.2.1. H1: Pancreas Volume Was Decreased in Patients with a New Diagnosis of T1D as Compared to Controls

We investigated whether patients with T1D showed a smaller pancreas at the time of clinical onset (Figure 1, H1). Because PV was dependent on the body weight (*p* < 0.050 at ANOVA), analyses were based on both PV and PVI as previously described [9, 15, 22, 40].

**Figure 2:**
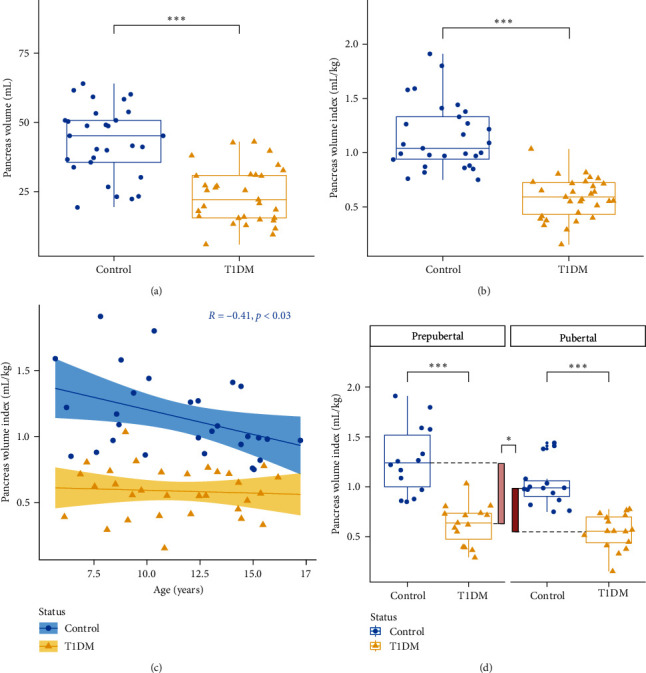
Pancreas volume parameters according to age and pubertal status in new-onset T1DM patients and age-, sex-, BMI-matched controls. MRI was performed closed to the diagnosis of T1D. Panels A-B represent pancreas volume (a) and pancreas index (b) in T1DM patients (*yellow*) and controls (*blue*). Panel C represents the evolution of pancreas index according to the age of the patient and the disease group (*yellow* = T1DM, *blue* = controls). *Shaded zone* around regression lines represent 95% confidence interval. Panel D represents the influence of pubertal status on pancreas reduction of volume between T1DM (*yellow*) and controls (*blue*). Vertical bars represents differences of median between prepubertal (*light orange*) and pubertal (*dark orange*) patients. Box plots display the median, 25^th^ and 75^th^ percentiles. The significance level is represented either by numerical values or signs ( ^*∗*^=*p* < 0.05,  ^*∗∗∗*^=*p* < 0.0001). Abbreviations: MRI, magnetic resonance imaging; R, spearman rho; T1DM, Type 1 diabetes mellitus.

Results from H1 are reported in Table 2. In brief, we observed a significant decrease of pancreas size in patients with new-onset T1D when compared to their matched nondiabetic controls (PV:−45%, PVI:−49%) (Figures 2(a) and 2(b), Table 1). PV was positively and significantly correlated with age in both T1D (*R* = 0.61) and control groups (*R* = 0.71). While PVI was significantly and negatively correlated with age in controls (*R* = −0.41), no correlation was observed in patients with T1D (Figure 2(c)).

Regarding the topography of pancreas destruction, PVR was similar in both groups (*p*=0.743) independently of the pubertal status (Table 1).

#### 3.2.2. H1: Patients with Prepubertal T1D Onset Has a More Pronounced Decrease of PVI

We investigated whether the decrease of PV varied according to the pubertal status and showed specific topographic patterns (diffuse or focal lesions) (Figure 1, H1). PV and PVI depended on the pubertal status and the anatomical area (*p* < 0.001) while being independent of sex (*p* > 0.05).

PV and PVI were significantly smaller in the T1D group regardless of the anatomical area and the pubertal status (Table S2). Prepubertal children had a significant reduction of PV as compared to pubertal patients in both patient groups while only a trend was observed for PVI in controls (Table S2). Indeed, patients with prepubertal onset of T1D had a greater reduction of PVI compared to the pubertal patients (PVI difference of mean ± SEM, 0.64 ± 0.09 vs. 0.44 ± 0.05 mL/kg; *p* < 0.001) corresponding to a decrease of −25% ± 2.1% in prepubertal T1D group (Figure 2(d)).

#### 3.2.3. H2: At T1D Onset, PV Parameters Were Correlated with Pancreatic Functions but Not with Clinical Phenotype

We investigated whether the reduction of PV at clinical onset (*Δ*) was associated with diabetes severity and pancreatic exocrine dysfunction. Results from H2 are reported in Table 2. Correlations between pancreatic MRI measures and parameters at *Δ* + 3 months for endocrine function, and at *Δ* for exocrine function test were assessed (Figure 1, H2). As pancreatic cell populations heterotopically distribute across the pancreas (e.g., most islets being located in pancreatic tail) [41, 42], correlations were assessed in both the whole pancreas and pancreatic subregions (head, body, and tail) (Table S3).

Characteristics of diabetes at clinical onset (i.e., HbA_1C_, C-peptide, bicarbonates, blood neutrophils absolute count, and glycemia) were not correlated with PV parameters or MRI parametric values, independently of pancreatic subregions (all *p* > 0.050, Table S3). Accordingly, patients presenting an inaugural episode of DKA (*n* = 9) did not show any difference in PV parameters compared to patients without DKA (*n* = 22) (*p* > 0.050, data not shown).

**Figure 3:**
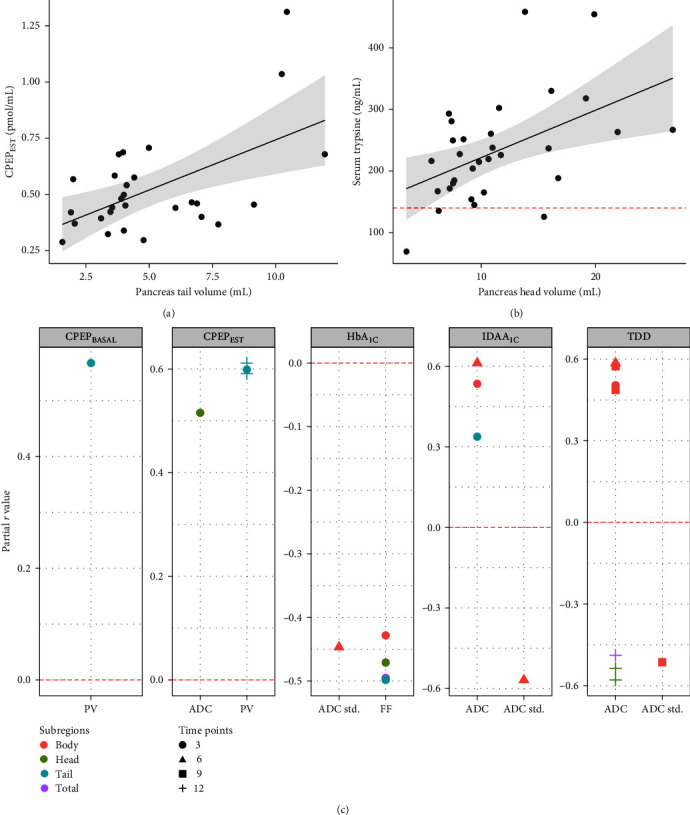
Pancreas MRI parameters at diagnosis correlate with residual pancreatic functions and improve prediction models of glucose homeostasis during the first year of T1D. Statistically significant topographic correlations between regional pancreatic subvolumes and residual B-cell secretion (calculated as described by Wentworth et al. [33]) (a) and exocrine function (b) with shaded zone around regression lines represent 95% confidence interval. The horizontal dashed red line in panel **B** represents the lower range of laboratory normality threshold. (c) Graphical representation of partial *r* values in multiparametric prediction models including MRI parameters (*x* axis). The horizontal dashed red line corresponds to *r* = 0. Dots are colored according to pancreatic subregions (PV_Body_ = red, PV_Head_ = green, PV_Tail_ = blue, PV_Total_ = purple) and shaped according to time from diagnosis (*Δ* + 3 months = round, *Δ* + 6 months = triangle, *Δ* + 9 months = square, *Δ* + 12 months = cross). The significance level is 0.05. Abbreviations: ADC, apparent diffusion coefficient; FF, fat fraction; HbA_1C_, glycated hemoglobin; CPEP_EST_, estimated C-peptide; CPEP_BASAL_, fasting C-peptide; IDAA_1C_, insulin dose-adjusted A1_C_; PV, pancreas volume; R, spearman rho; ADC std., standard deviation of ADC; T1D, type 1 diabetes; TDD, total insulin daily dose.

Correlations between PV parameters and residual *β*-cell secretion (i.e., CPEP_EST_ or CPEP_BASAL_, *n* = 29) or glycemic control (i.e., clinical parameters, *n* = 31) measured close to diagnosis were also evaluated. While CPEP_EST_ and CPEP_BASAL_ were not correlated with PV and PVI from the whole pancreas (all *p* > 0.050, Table S3), both residual secretion estimates significantly correlated with pancreatic tail volume (PV_Tail_) (*R* = 0.55; *R* = 0.50, respectively) (Figure 3(a)) but not with other pancreatic subregions (PV_Head_ and PV_Body_) (Table S3). No correlation was found between clinical parameters at *Δ* + 3 months (IDAA_1C_, IDD, or HbA_1C_) and PV parameters, independently of pancreatic subregions (all *p* > 0.050) (Table S3).

As pancreas mass is mostly composed of acinar cells, we investigated whether circulating markers of exocrine function (fasting serum lipase and trypsinogen, *n* = 31) measured around MRI scan (*Δ*) witnessed the reduction of PV in patients with T1D. Most of the trypsinogen values were within the laboratory ranges (*n* = 28 [90%]). Serum trypsinogen and lipase were significantly correlated with both PV and PVI of the whole pancreas (*R* between 0.45 and 0.58) (Figure S2, Table S3). Topographical pancreatic analysis showed a specific significant correlation between trypsinogen and PV_Head_ (*R* = 0.49) (Figure 3(b)) but not with other pancreatic subvolumes (Table S3).

#### 3.2.4. H3: Pancreas Volume Parameters Improved Prediction Models of Endocrine Function during the First Year after T1D Onset

We performed an explanatory study to investigate whether multiparametric models including PV parameters and parametric MRI values (ADC and FF) at diagnosis predicted pancreatic endocrine functions during the first year of T1D (Figure 1, H3). Clinical parameters (HbA_1C_, IDAA_1C_, and IDD) were collected at *Δ* + 3 months (*n* = 31), *Δ* + 6 months (*n* = 29), *Δ* + 9 months (*n* = 28), and *Δ* + 12 months (*n* = 29). Residual *β*-cell secretion estimates (CPEP_EST_ and CPEP_BASAL_) were collected at *Δ* + 3 months (*n* = 29) and *Δ* + 12 months (*n* = 25). Results from H3 are reported in Table 2 with most interesting biparametric predictive models being summarized in Table S4.

In brief, pancreas FF in both whole pancreas and pancreas head significantly predicted HbA_1C_ levels at *Δ* + 3 months (Table S4). Also, biparametric models including PV_Tail_ and sex substantially predicted CPEP_EST_ (*R* = 0.65) and CPEP_BASAL_ (*R* = 0.69) at *Δ* + 3 months, and CPEP_EST_ (*R* = 0.42) at *Δ* + 12 months (Table S4). Finally, several models including pancreas diffusion coefficient (i.e., ADC) significantly predicted IDAA_1C_ and TDD at *Δ* + 3, *Δ* + 6, and *Δ* + 9 months (Table S4). For example, biparametric models including serum trypsinogen and mean ADC_Body_ significantly predicted both IDAA_1C_ at *Δ* + 3 months, and TDD at *Δ* + 3, *Δ* + 6, and *Δ* + 9 months with an *R* of 0.64, 0.62, 0.67, and 0.59, respectively (Figure 3(c), Table S4).

#### 3.2.5. H4: Longitudinal Evolution of PVI Differed between Patients and Did Not Reflect the Evolution of Glucose Homeostasis

We investigated the evolution of PV parameters as well as parametric MRI values at 1-year postdiagnosis. Twenty-two patients (73%) underwent a second imaging session after a mean duration of 14 ± 2.6 months. PV parameters were normalized for 12 months considering a linear decline or increase (PV^T12^ and PVI^T12^) (Figure 1, H4).

**Figure 4:**
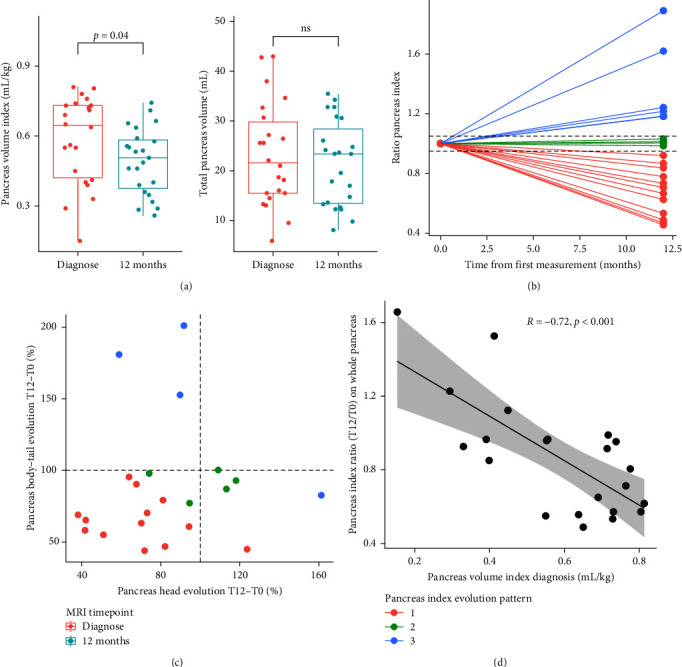
Morphological evolution of pancreas during the first year after T1D onset. (a) Differences in pancreas index (left panel) and pancreas volume (right panel) in T1D patients. (b) Visualization of relative evolution of PVI during the first year of T1D (PVI^T12^/PVI^T0^) with PVI^_T12_^ being normalized at *Δ* + 12 months considering a linear evolution. Horizontal dashed lines represent pancreas index ratio values of 1.05 (upper) and 0.95 (lower) delineating PVI evolution pattern (i.e., increasing (blue dots), stabilizing (green dots) or decreasing (red dots)). (c) Topographic visualization of pancreas volume evolution according to body–tail (*y* axis) and head (*x* axis) subregions. Colors of the dots represent the PVI evolution pattern (i.e., PVI^T12^/PVI^T0^). Dashed lines represent a ratio of 100% (i.e., no change) with value below demonstrating a decrease of PV during the first year of T1D. (d) Linear regression with 95% CI bands (shaded zone) between PVI at diagnosis and PVI ratio (T12/T0). *R* represent the regression coefficient of Spearman. Significance level was *p* value < 0.05 for all analysis. Abbreviations: CI, confidence interval; PVI, pancreas index; T1D, type 1 diabetes.

We observed a significant decrease of whole pancreas PVI of −0.09 ± 0.16 mL/kg during the first year after diagnosis, but not PV (Table 1, Figure 4(a)). A large variability between patients was observed in the longitudinal evolution of PVI (i.e., PVI^T12^/PVI^T0^ ratio): respectively, 4 (18%), 5 (23%), and 13 (59%) patients increased (PVI^T12^/PVI^T0^ > 1.05), stabilized (0.95 < PVI^T12^/PVI^T0^ < 1.05), or decreased (PVI^T12^/PVI^T0^ < 0.95) their PVI during the first year following T1D onset (Figure 4(b)). For each patient, the trends of PVI evolution were globally homogenous within pancreatic subregions (Figure 4(c)). When we investigated the phenotypic characteristics of these three groups, we found that patients significantly differed by their PVI at first MRI scan but had similar clinical characteristics at diagnosis (either in HbA_1C_, HCO3^−^, C-peptide, neutrophils count, plasmatic glycemia or in pubertal status) and during the first year longitudinal follow-up (either in HbA_1C_, IDAA_1C_, TDD or in serum lipase at *Δ* + 3, *Δ* + 6, *Δ* + 9, and *Δ* + 12) (*p* > 0.050, data not shown). The reduction of PVI during the first year after T1D onset (PVI^T12^/PVI^T0^) was indeed inversely and significantly correlated with PVI^T0^ (*R* = −0.72) (Figure 4(d)). The analysis of ADC maps showed a small but significant decrease in whole pancreatic diffusion at *Δ* + 12 months (ADC^TO−T12^ = −110 mm^2^/s). No significant change in FF maps maps were observed between the two timepoints.

#### 3.2.6. H4: Evolution of MRI Parameters during the First Year Postdiagnosis Were not Correlated with Pancreatic Endocrine Functions though Topographic Correlations Persisted

As structural modifications of pancreas might affect *β* cells, we investigated whether differences observed in parameters measured by MRI (diffusion and FF or volume parameters) between T0 and T12 witnessed the change of pancreatic functions across the first year postdiagnosis (Figure 1, H4).

Results are reported in Table S5. No significant correlation was found between the absolute or relative differences of volume parameters and parameters of glucose homeostasis (CPEPEST, HbA_1C_, IDAA_1C_, or TDD). Differences in diffusion or FF were not correlated with differences in endocrine function parameters, regardless of the pancreatic subregions studied.

We finally investigated whether topographical correlations between residual *β*-cell secretion (CPEP_EST_, *n* = 21) and volume parameters of pancreatic subregions around diagnosis were still significant at *Δ* + 12 months. PV_Tail_ remained correlated with CPEP_EST_ measured at +12 months (*R* = 0.57, *p*=0.008) but not with clinical parameters of glucose homeostasis (IDAA_1C_, TDD, or HbA_1C_; *p* > 0.050) (Figure S3).

## 4. Discussion

The main results of this study are the following ones. H1: the pancreas of pediatric patients with new-onset was homogeneously smaller compared to controls. Patients with prepubertal T1D onset had a more important decrease of PVI compared to the pubertal group. H2: Topographic correlations were observed between the volumes of pancreas subregions (PV_Tail_ and PV_Head_), and residual *β*-cell secretion and exocrine function, respectively, measured around T1D diagnosis. Topographical correlation with residual *β*-cell secretion persisted at *Δ* + 12 months. H3: the integration of PV parameters, ADC, or FF values measured at diagnosis into biparametric models improved the prediction of glucose homeostasis during the first year after diagnosis. H4: the longitudinal evolution of PVI after T1D diagnosis varied among patients, either increasing or decreasing. A significant and inverse correlation with PVI at diagnosis were observed (*R* = −0.72), but not with routinely assessed parameters at diagnosis or during the follow-up (e.g., HbA_1C_, IDAA_1C_, and IDD).

It remains highly debated as to whether the reduction of pancreas size and subclinical exocrine dysfunction result from a primary event involving the whole pancreas or from the primary loss of *β* cells (due to loss of insulin trophic factor) [7–9, 43–46]. Our data support the first hypothesis, showing a homogeneous distribution of both PV reduction and parametric MRI values across all pancreatic subregions. Our results agreed with *in vivo* and histological findings from previous reports. Imaging studies of T1D pancreas showed similar right and left side decrease of size [40] with homogeneous distribution of pancreas inflammation in most new-onset T1D patients (*n* = 6/11) [22]. Histological evaluation of pancreases from patients with T1D corroborated these findings as most cell populations from both innate and adaptative immunity showed diffuse tissue infiltration, independently of endocrine or exocrine compartments [43, 47, 48]. These results together support that the reduction of PV in patients with T1D most likely results from a phenomenon involving the whole organ rather than a specific pancreatic subregion. Nonetheless, our team and others observed a high-interpatient variability in pancreas size reduction among patients with new-onset T1D [9, 10, 15, 22]. Specifying these observations, a recent study observed an alteration of pancreas shape in patients with T1D [25].

Over the past years, there has been compelling evidence that patients with T1D presented both clinical and histological heterogeneity [2, 16–19, 49, 50]. Our results showed a puberty-related pattern characterized by a highest reduction of PVI in children with prepubertal T1D onset. Supporting age-related disparities, distinct histological patterns (in the endocrine compartment), and specific clinical features were observed in children with young-onset T1D (i.e., <7 years old) compared to the older onset (>12 years old). These were recently regrouped under the term “endotypes” [2, 17–20, 49]. In that regard, our findings give new insights into age-related differences in pancreas morphology and support a more aggressive disease in children with young T1D-onset [2, 17].

Interpatient variability was also observed in the evolution of PV after T1D onset (i.e., three patterns). Our results suggested that patients with the highest PVI at diagnosis had a more important reduction of their PVI after onset though no association with clinical routine parameters of glucose homeostasis were observed. These results were consistent with a recent study that demonstrated a global reduction of PVI during the first year after T1D onset (−0.0084 mL/kg/month) with high-interpatient variability and PVI trajectories [15]. Interestingly, a recent study showed both a longitudinal reduction of PV and an alteration of pancreas shape after T1D onset that were poorly or not associated with glucose homeostasis parameters [25]. These observations together suggest that PV parameters have variable evolution after T1D onset that is not captured by the current markers of diabetes follow-up such as glucose homeostasis [9, 10].

This study also showed that whole PV were correlated with markers of exocrine (serum lipase and trypsinogen) but not endocrine function. Similar results were reported by most studies for exocrine function markers [26, 29, 51].

Conclusion about correlations with endocrine markers (e.g., residual *β*-cell secretion, TDD, and HbA_1C_) remains difficult to establish due to discrepant results reported in literature [10, 15, 27, 51–53] with high variability in the evaluation methods and the T1D population studied.

This study showed moderate topographic correlations between both endocrine and exocrine functions, and pancreatic subvolumes at diagnosis (PV_Tail_ and PV_Head_) and *Δ* + 12 months (PV_Tail_). Endocrine pancreatic cells (e.g., islets number and *β*-cells area) distribute heterotopically across the pancreas, being mostly located in the pancreas body and tail in the healthy patients [41, 42]. Supporting these histological descriptions, the incidence of new-onset diabetes mellitus was higher following a distal pancreatotomy compared to the central or proximal pancreatotomy [54, 55] while pancreas head resection did not alter glucose homeostasis at 12 months [55, 56]. Evidence in patients with T1D remains more arguable. Nonetheless, recent reports in patients with T1D suggested an increased loss of *β* cells in the dorsal lobe of the pancreas [57] while another demonstrated similar *β*-cell mass in both pancreas tail and body [47]. Topographic analysis of the pancreas by MRI (including subvolumes and shape) may be an additional noninvasive tool to improve the prediction of pancreatic functions evolution during the first year of T1D or even the progression to Stage 3 [25].

Our study has several strengths. It is the first MRI study that evaluates the topography of PV reduction and the presence of age-related differences in the exocrine pancreas in a cohort including 50% of prepubertal patients. Longitudinal assessment of MRI parameters (volume, ADC, and FF) and pancreatic functions allowed both cross-sectional comparisons and the identification of potential predictive models of pancreatic functions.

This study has also several limitations. MRI was performed in a nonfasting state to decrease the risk of hypoglycemia during the imaging session. A recent study demonstrated no influence of the prandial state on pancreatic measurements [23]. As there is no clear consensus on the anatomical limits between the pancreas body and tail, the limits described in previous studies were applied strictly [38, 39]. Then the segmentation was reviewed during a final consensus reading with a radiologist expert, thus limiting a potential influence of the nonfasting state on MRI measures. The size of the studied cohort was small. As adjusting the significance level of the statistical tests is not recommended in such a case, *p*-values were not corrected when performing multiple comparisons for the same hypothesis [58]. Therefore, hypotheses generated from the multiparametric regression models should be considered with caution and further tested on a new independent and larger cohort. This study however regroups one of the biggest cohorts of pediatric new-onset T1D patients assessing both pancreas imaging and pancreatic functions [9].

In conclusion, children with new-onset T1D demonstrated an age-related homogenous reduction of PV parameters with prepubertal patients having increased volume reduction compared to controls. Topographic correlations between pancreatic subregion parameters and their respective residual functions, both early after the diagnosis and after the first year of diabetes, were observed. Volume, FF, and apparent diffusion coefficient measures from MRI may contribute in predicting the endocrine function during the first year of T1D, especially when analyzed topographically. Longitudinal evolution of PV after clinical T1D onset was correlated with pancreas size at diagnosis but not with common markers of glucose homeostasis. We believe that *in situ* analysis of T1D pancreas using MRI might improve the understanding of the heterogeneity observed within patients and provide additional noninvasive imaging markers of the pancreatic functions.

## Figures and Tables

**Table 1:** Characteristics of the population.

	Global	Controls	T1D	*p*-Value* ^*∗*^*
	(*N* = 60)	(*N* = 29)	(*N* = 31)	
Distribution				
Age (years)	11 ± 3.1	12 ± 3.3	11 ± 2.9	0.70^†^
Height (*Z*-score)	−0.15 ± 1.12	−0.37 ± 1.05	0.04 ± 1.12	0.22^†^
Weight (*Z*-score)	−0.16 ± 1.01	−0.32 ± 0.91	0.00 ± 1.05	0.24^†^
BMI (*Z*-score)	0.02 ± 1.12	−0.14 ± 1.12	0.12 ± 1.08	0.36^†^
Sex—Female no. (%)	25 (42)	12 (41)	13 (43)	>0.99^‡^
Prepuber no. (%)	29 (48)	15 (52)	14 (45)	>0.99^‡^
Baseline diabetes characteristics				
HbA_1C_ (%)	NA	NA	13 ± 2.0	NA
Presence of ketoacidosis no. (%)	NA	NA	9.0 (29)	NA
Glycemia (mg/dL)	NA	NA	540 ± 196	NA
Weight loss (%)	NA	NA	14 ± 8.2	NA
Diabetes duration at imaging (days)	NA	NA	40 ± 37	NA
Pancreas volume parameters at diagnosis				
PV (mL)	33 ± 15	43.1 ± 12.7	23.4 ± 10.1	<0.001^†^
Pancreas volume index (mL/kg ^*∗*^)	0.9 ± 0.4	1.2 ± 0.3	0.6 ± 0.2	<0.001^†^
Pancreas volume ratio	1.4 ± 0.6	1.5 ± 0.4	1.4 ± 0.7	0.74^†^
Glycemic control^||^				
Remitters no. (%)	NA	NA	20 (66)	NA
HbA_1C_ (%)	NA	NA	6.0 ± 0.7	NA
Insulin doses (IU/kg/day)	NA	NA	0.6 ± 0.3	NA
IDAA_1C_	NA	NA	8.5 ± 1.4	NA
CPEP_EST_^§^	NA	NA	0.5 ± 0.3	NA
Exocrine function				
Trypsinogen (ng/mL)	NA	NA	234 ± 85.0	NA
Pancreas volume parameters at 12 months				
Pancreas volume (mL)	NA	NA	22 ± 8.2	NA
Pancreas volume index (mL/kg)	NA	NA	0.5 ± 0.15	NA

Plus–minus values are means ± SD. Percentages may not total to 100 because of rounding.  ^*∗*^*p*-Value calculated between type 1 diabetes and control group. Results were considered as significant when under 0.05. ^†^Student *t*-test, ^‡^*χ*^2^, ^||^Parameters evaluated at 3 months after diagnosis, ^¶^Wilcoxon-test ^§^calculated as described by Wentworth et al. [33]. Abbreviations: BMI, body mass index; HbA_1C_, glycated hemoglobin level; IDAA_1C_, insulin dose adjusted A_1C_; NA, not applicable; T1D, type 1 diabetes.

**Table 2:** Summary of principal hypotheses of the study.

***H1: Comparison of PV parameters between patients with T1D and controls***
Patients with T1D have smaller pancreas than controls Patients with prepubertal T1D onset have more pronouced reduction of PVI than postpubertal
***H2: Correlations between PV parameters and pancreas functions***
Whole PV and PVI correlated with exocrine but not markers of diabetes at clinical onset or residual endocrine function at +3 months Topographic correlations between PV and residual B-cell secretion and exocrine function
***H3: Prediction of glucose homeostasis evolution using pancreas MRI parameters at diagnosis***
Biparametric models including MRI parameters improved prediction of endocrine function in the first year following T1D onset
***H4: Evolution of MRI parameters after T1D onset and pancreas function***
PVI decreases during first year postdiagnosis but interpatient variability exists (3 patterns) Reduction of PVI^T12^ was inversely correlated with PVI^TO^ Evolution of MRI parameters between T_0_ and T_12_ does not correlated with pancreas function though topographic correlations remains (i.e., PV_Tail_ and CPEP_EST_)

Abbreviations: MRI, magnetic resonance imaging; PV, pancreas volume; PVI, pancreas volume index; T1D, type 1 diabetes.

## Data Availability

The authors will make the raw data supporting this article's conclusion available upon request and acceptance.

## Supplementary Materials

Supplementary Materials Table S1: summary of technical parameters of MRI sequences. Table S2: pancreas volume and pancreas index measurements per status and per puberty. Table S3: correlations between pancreas volume parameters and pancreatic functions. Table S4: summary of most interesting bivariate predictive models of glucose homeostasis during the first year of T1D. Table S5: correlations between absolute difference of PV and PVI between T0 and T12, and differences between pancreas endocrine function. Figure S1: visualization of pancreas subregions delimitations. Figure S2: linear regressions between pancreas volume parameters and serum trypsinogen at clinical onset of type 1 diabetes. Figure S3: linear regressions between pancreas volume parameters and CPEP_EST_ at 12 months postdiagnosis.
