# Supplementary material for: Pancreas Imaging of Children with Type 1 Diabetes Reveals New Patterns and Correlations with Pancreatic Functions
Source: Pediatr Diabetes. 2023 Sep 22;2023:3295812. doi: 10.1155/2023/3295812 (PMC12017098; doi:10.1155/2023/3295812)
Supplement: Supplementary Materials — Table S1: summary of technical parameters of MRI sequences. Table S2: pancreas volume and pancreas index measurements per status and per puberty. Table S3: correlations between pancreas volume parameters and pancreatic functions. Table S4: summary of most interesting bivariate predictive models of glucose homeostasis during the first year of T1D. Table S5: correlations between absolute difference of PV and PVI between T0 and T12, and differences between pancreas endocrine function. Figure S1: visualization of pancreas subregions delimitations. Figure S2: linear regressions between pancreas volume parameters and serum trypsinogen at clinical onset of type 1 diabetes. Figure S3: linear regressions between pancreas volume parameters and CPEPEST at 12 months postdiagnosis. [file 3295812.f1.doc]

**Supplementary material**

**Supplementary Table 1.** **MRI acquisition parameters.**

| Sequence | Field-of-view (Frequency x Phase, cm) | Matrix (Frequency x Phase) | Slice Thickness/gap (mm) | TE/TR  (ms) | #slices | Breath-holds | Total duration |
| --- | --- | --- | --- | --- | --- | --- | --- |
| T2-SSFSE** | 30 x 30 | 400 x 224 | 3/0 | 85/525 | 40 | 2 | 0 min 22 s |
| T1-SPGR  (LAVA-Flex)** | 32 x 29 | 292 x 256 | 3.4/0 | 1.1-2.2/4.1 | 84  (168 reconstructed) | 1 | 0 min 13 s |
| DWI^¶^ | 36 x 36 | 128 x 160 | 4/1 | 50/7058* | 40 | 0 | 3 min 04 s* |
| Multi-echo GRE  (IDEAL-IQ) | 44 x 44 | 160 x 160 | 5/0 | 0.9-4.3/5.8 | 56 | 1 | 0 min 16 s |

*These values of TR and acquisition duration are hypothetical given that they were dependent on the respiratory rate. The given values are for a respiratory rate of 17/min. The body RF-coil was used for transmission and multi-array coils (in the table and flexible AIR coils over the patient) for reception. ** Flip angle 12°.^¶^ Two averages were used for diffusion encoded images b=50 s/mm² and six for b=800 s/mm² and parallel imaging (ASSET/SENSE) with an acceleration factor of 2. To minimize breathing-induced motion, respiratory gating was employed with a navigator placed at the top of the liver dome.

TE: time of echo; TR: time of repetition.

**Supplementary Table 2.** Pancreas volume (PV) and pancreas volume index (PVI) measurements *per* status and *per* puberty.

| **Pancreas volume indices** | | **Control** | |  |
| --- | --- | --- | --- | --- |
|  |  | **Puber** | **Prepuber** | **difference** |
| PV (mL) | Head | 20.7 [18.5; 22.9] | 15.1 [11.6; 18.5] | +5.62 [+1.75; +9.51]**^‡^ |
|  | Body+Tail | 29.6 [25.3; 34.0] | 20.3 [17.0; 23.5] | +9.39 [+4.15; +14.6]***^‡^ |
|  | Whole pancreas | 50.4 [45.1; 55.6] | 35.4 [29.0; 41.8] | +15.0 [+7.12; +22.8]***^‡^ |
| PI (mL/kg) | Head | 0.43 [0.36; 0.50] | 0.54 [0.43; 0.65] | - |
|  | Body+Tail | 0.60 [0.52; 0.67] | 0.73 [0.63; 0.83] | -0.13 [-0.25; -0.02]*^‡^ |
|  | Whole pancreas | 0.99 [0.89; 1.07] | 1.27 [1.07; 1.47] | - |
| **Pancreas volume indices** | | **T1D** | |  |
|  |  | **Puber** | **Prepuber** | **difference** |
| PV (mL) | Head | 13.3 [10.0; 16.5] | 8.13 [7.29; 9.70] | +5.45 [+0.69; .7.84]*^†^ |
|  | Body+Tail | 18.01 [14.2; 21.8] | 9.36 [7.38; 14.9] | +8.12 [+0.98; +11.7]*^†^ |
|  | Whole pancreas | 27.8 [22.6; 33.0] | 15.5 [13.6; 24.8] | +10.2 [+3.50; +15.8]*^†^ |
| PI (mL/kg) | Head | 0.26 [0.20; 0.32] | 0.31 [0.26; 0.37] | - |
|  | Body+Tail | 0.35 [0.29; 0.42] | 0.39 [0.30; 0.47] | - |
|  | Whole pancreas | 0.55 [0.46; 0.64] | 0.63 [0.52; 0.74] | - |
|  |  | **difference** | **difference** |  |
|  |  | +7.45 [+3.62; +11.3]***^‡^ | +5.33 [+1.10; +11.1]**^†^ |  |
|  |  | +11.6 [+6.12; +17.1]***^‡^ | +8.47 [+3.36; +13.4]**^†^ |  |
|  |  | +22.6 [+15.5; +29.7]***^‡^ | +15.2 [+7.85; +26.3]***^†^ |  |
|  |  | +0.17 [+0.08; +0.25]***^‡^ | +0.23 [+0.10; +0.35]**^‡^ |  |
|  |  | +0.25 [+0.15; +0.34]***^‡^ | +0.34 [+0.22; +0.46]***^‡^ |  |
|  |  | +0.44 [+0.30; +0.62]***^†^ | +0.64 [+0.43; +0.86]***^‡^ |  |

| *Differences are calculated as follows: Pubertal - Prepubertal, and Control – T1D (expressed in ml for PV and in % for PVI)* |
| --- |
| *^‡^Mean difference from t-test, ^†^Median difference from Mann-Withney U test. Abbreviations : PVI= pancreas volume index, PV = pancreas volume, T1D = type 1 diabetes. The level of significance of the differences is represented as follows: p<0.05 (*), p<0.01 (**), p<0.001 (***).* |

**Supplementary Table 3.** Correlations between pancreas volume parameters and, diabetes characteristics at onset and pancreatic functions

*Correlations were calculated using Spearman method. ^§^ measured at 3 months postdiagnosis. * Calculated according to Wentworth et al* (1) *Abbreviations :* R: Spearman *rho*

**Supplementary Table 4.** Summary of most interesting bivariate predictive models of glucose homeostasis during the first year of type 1 diabetes.

| ***Glucose homeostasis parameter*** | ***Time-point*** | ***Parameter 1*** | ***Parameter 2*** | ***Multiple correlation coefficient*** | ***p value*** | ***Clinical meaning*** |
| --- | --- | --- | --- | --- | --- | --- |
|  | ***(months)*** |  |  | ***(R)*** |  |  |
|  |  |  |  |  |  |  |
| HbA_1C_ | 3 | mean FF_HEAD_ | Puberty | 0.71 | 0.0001 | Prepubertal and lower pancreatic FF predicted higher HbA_1C_ at 3 months |
| HbA_1C_ | 3 | mean FF_WHOLE_ | Puberty | 0.65 | 0.0005 | Prepubertal and lower pancreatic FF predicted higher HbA_1C_ at 3 months |
| CPEP_BASAL_ | 3 | PV_TAIL_ | Sex | 0.65 | 0.0007 | Bigger PV_TAIL_ and male gender predict higher CPEPBASAL at 3 months |
| CPEP_EST_ | 3 | mean ADC_HEAD_ | Puberty | 0.65 | 0.0008 | Postpubertal and increased ADC predict higher CPEP_EST_ at 3 months |
| IDAA_1C_ | 3 | mean ADC_BODY_ | Trypsinogen | 0.64 | 0.0008 | Increase of ADC and Trypsinogen predict higher IDAA_1C_ at 3 months |
| TDD | 3 | mean ADC_BODY_ | Trypsinogen | 0.62 | 0.0014 | Increase of ADC_mean_ and trypsinogen predict higher TDD at 3 months |
| CPEP_EST_ | 3 | PV_TAIL_ | Sex | 0.62 | 0.002 | Bigger PV_TAIL_ and male gender predict higher CPEPEST at 3 months |
| IDAA_1C_ | 6 | mean ADC_BODY_ | STD ADC_BODY_ | 0.69 | 0.0004 | Increase of ADC_mean_ with low ADC SD predict higher IDAA1C at 6 months |
| TDD | 6 | mean ADC_BODY_ | Trypsinogen | 0.67 | 0.0006 | Increase of ADC_mean_ and trypsinogen predict higher TDD at 6 months |
| TDD | 9 | mean ADC_BODY_ | Trypsinogen | 0.58 | 0.005 | Increase of ADC_mean_ and trypsinogen predict higher TDD at 9 months |
| CPEP_EST_ | 12 | PV_TAIL_ | Sex | 0.65 | 0.003 | Bigger PV_TAIL_ and male gender predict higher CPEP_EST_ at 12 months |

**Supplementary Table 5 :** Correlations between absolute difference of Pancreas Volume and Pancreas Volume Index between T^0^ and T^12^, and differences between pancreas endocrine function.

*Correlations were calculated using Spearman method. ^§^ Differences were calculated as Parameter^T12^ - Parameter^T0^. * Calculated according to Wentworth et al* (1) *Abbreviations p= p-value.*

**Supplementary Figure 1**


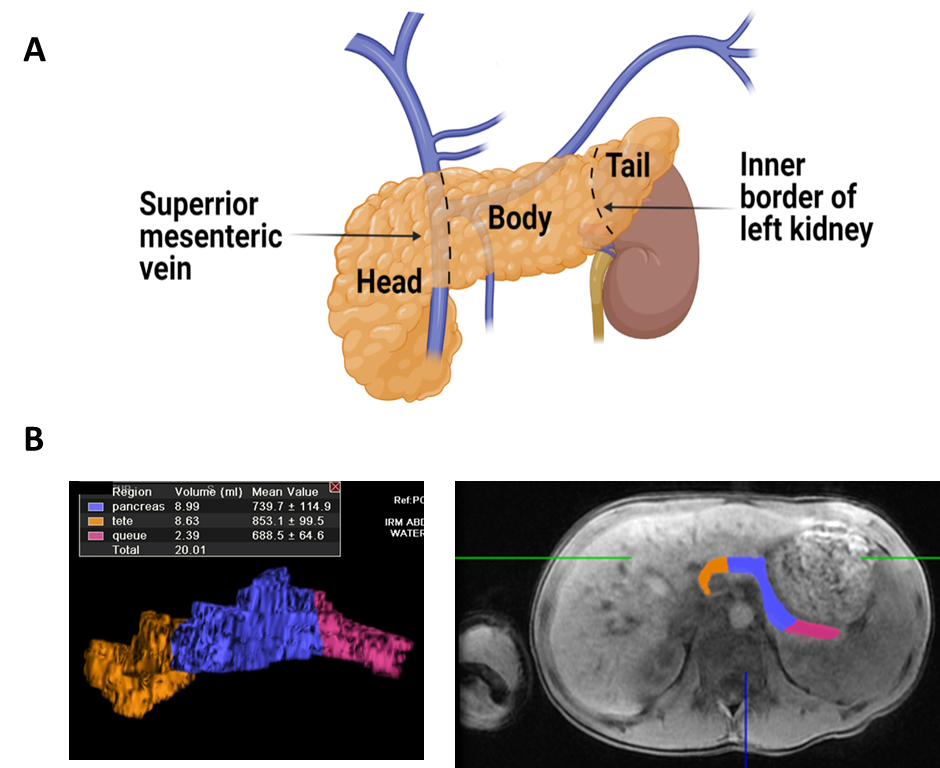


**Supplementary Figure 1:** Visualization of pancreas subregions delimitations. (**A**) Graphical/Schematic representation of pancreas division into three subregions (i.e., head, body and tail) according to predefined anatomical limits (*dashed lines*). (**B**) Pancreas subregions volumetry (*left panel*) of a nine-years-old girl with new-onset type 1 diabetes measured using Vitrea^TM^ software on anatomic MRI sequence (*right panel*). MRI: magnetic resonance imaging.

**Supplementary Figure 2**


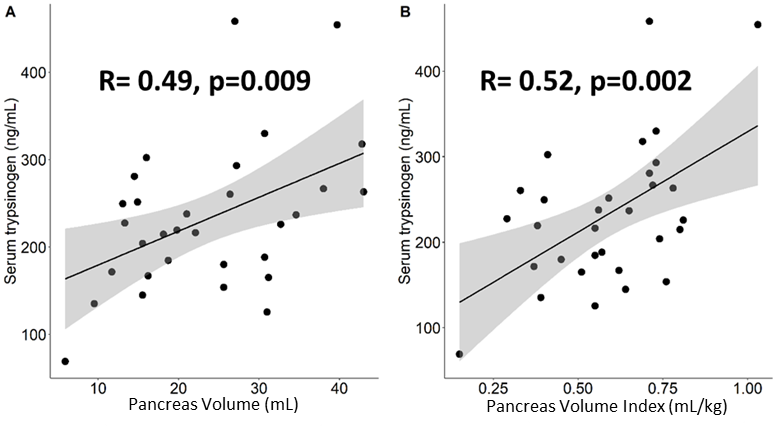


**Supplementary Figure 2:** Linear regressions between PV (**A**) and PVI (**B**), and serum trypsinogen at clinical onset of T1D. Shaded zone around regression lines represent 95% confidence interval. PVI: pancreas volume index; PV: pancreas volume; R: Spearman *rho*; T1D: type 1 diabetes.

**Supplementary Figure 3**


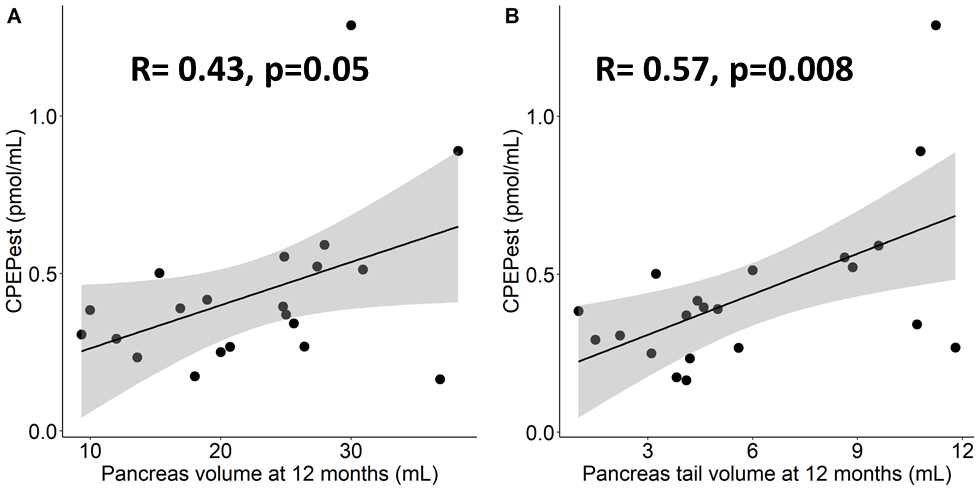


**Supplementary Figure 3:** Linear regressions between PV (**A**) and PV_TAIL_ (**B**), and CPEP_EST_ at 12 months postdiagnosis. Shaded zone around regression lines represent 95% confidence interval. PV: pancreas volume; R: Spearman *rho*. CPEP_EST_ was calculated according to Wentworth *et al* (1).
